# Effective cleaning of endoscopic lenses to achieve visual clarity for minimally invasive abdominopelvic surgery: a systematic review

**DOI:** 10.1007/s00464-021-08519-6

**Published:** 2021-05-07

**Authors:** Ahmad Nabeel, Salman K. Al-Sabah, Hutan Ashrafian

**Affiliations:** 1 Department of Surgery and Cancer, Imperial College London, London, United Kingdom; 2 Institute of Global Health Innovation, Imperial College London, London, United Kingdom; 3 Department of Surgery, Jaber Al-Ahmad Al-Sabah Hospital, Kuwait City, Kuwait; 4 Department of Surgery, Faculty of Medicine, Health Sciences Centre, Kuwait University, Kuwait City, Kuwait

**Keywords:** Minimally invasive surgery, Lens cleaning, Endoscopy, Abdominopelvic surgery, Systematic review

## Abstract

**Objective:**

To review the recently available interventions to achieve optimal visual clarity in laparoscopic abdominopelvic surgery compared to conventional cleaning alternatives.

**Summary background data:**

Currently, there is no consensus on the most effective method for the cleaning of endoscopic lenses used in minimally invasive abdominopelvic surgery.

**Methods:**

Literature searching for articles relevant to answering a predefined research question was performed in December 2019 and involved searching of the electronic databases of MEDLINE, the Cochrane Registry, and EMBASE. Basic search terms were derived using the PICO (population, intervention, comparator and outcomes) framework and through a scoping search of literature via MEDLINE. A manual search of Google Scholar and citation screening of eligible studies was also performed to ensure the identification and inclusion of all pertinent studies to address the research question.

**Results:**

Among conventional and readily available methods, the most effective approaches involved heated sterile water, heating of laparoscope lenses, and surfactant solutions, including FRED and Ultra-Stop, while evaluations of all novel devices and methods were more effective than controls, which included lens wiping systems and air and carbon dioxide flow systems. While the former surgical techniques were consistently associated with superior lens cleaning ability and/or defogging capability and subsequent optical clarity of images within the surgical field, no methods conferred any meaningful effects upon other clinically important outcomes, such as operative time, costs, complication rates and length of stay, suggesting that decision making concerning the selection of lens cleaning method/device should suit the preferences of the instrument operator and/or the responsible surgeon.

**Conclusions:**

We demonstrated that a range of endoscopic lens cleaning methods and devices can be used to achieve sufficient optical clarity of the laparoscopic surgical field through either preventing lenses from fogging and/or facilitating the inter-operative cleaning of fouled lenses. Despite the various methods evaluated in this review, there were no significant differences in complication rates between the intervention and control groups.

A laparoscope is an optical rod-shaped device that allows surgeons to see the anatomical structures of the body using a camera system that is inserted through a small surgical incision. The captured images are then processed in real-time by a video unit and projected onto a specific screen for surgeons to view [1].

The utilisation of laparoscopes in surgery allows surgeons to visualise the viscera without having to make significantly large incisions which, by itself, provides benefits to patient outcomes, such as reduced blood loss, early hospital discharge, fewer surgical site infections, and enhanced aesthetic results. On the other hand, technical drawbacks of laparoscopy include attenuated tactile sensation when compared to open surgery, difficulty accessing hard-to-reach areas, limited wrist articulation, reduced depth perception, and poor off-screen awareness of non-visible tissues. This forces surgeons to heavily depend upon their vision [2]. Despite the technological advancement in surgical optics like 3D vision and 4 K resolution cameras, laparoscopic lens fouling is still an ongoing problem that is not easy to prevent or resolve peri-operatively. Conventional cleaning methods normally halt surgery, requiring the retraction of the scope out of the patient’s body via the port site to permit manual cleaning of the lens. This poses performance and safety risks. Ideally, there would be a way to clean the lens or maintain its clarity without having to disrupt surgery and withdraw the laparoscope from the patient and without disturbing the concentration of operating surgeons.

This research aimed to systematically review the recent and readily available interventions designed to achieve optimal visual clarity in laparoscopic abdominopelvic surgery. Such evidence should advance understanding into the optimal lens cleaning methods that could be used to benefit laparoscopic surgery in future practice.

## Methods

A systematic review was conducted in accordance with the methods and procedures defined by the Cochrane Collaboration and within the preferred reporting items for systematic reviews and meta-analyses (PRISMA), in order to address the following research question [3, 4].

What are the most effective methods for cleaning endoscopic lenses to achieve optimal visual clarity for the purposes of minimally invasive abdominopelvic surgery?

### Search strategy

Literature searching for articles relevant to answering the former research question was performed in December 2019 and involved searching of the electronic databases of MEDLINE, the Cochrane Registry, and EMBASE as this combination has been demonstrated to have high study retrieval accuracy [5]. Basic search terms were derived using the PICO (population, intervention, comparator and outcomes) framework (Table 1) and through a scoping search of literature via MEDLINE. The final terms applied to database searching along with any relevant syntax are summarised in Table 2. A manual search of Google Scholar and citation screening of eligible studies was also performed to ensure the identification and inclusion of all pertinent studies to address the research question. Notably, searching using the initial terms was ineffective and thus, a broader search was employed using the terms in column three of Table 2.

**Table 1:** PICO framework to guide the review

PICO	
Population	Patients or experimental models receiving laparoscopic abdominopelvic surgery
Intervention	Endoscope lens cleaning method/device
Comparator	Other endoscope lens cleaning methods/devices
Outcomes	Optical clarity of the surgical field
	Number of lens cleaning attempts
	Lens cleaning duration
	Other surgical outcomes

**Table 2:** Search strategy

Electronic database	Initial search terms/syntax	Broad search terms
MEDLINE	“Endoscope” OR “laparoscope” AND “lens” OR “lenses” AND “clean” OR “cleanliness” OR “decontaminate” OR “wash” AND “optical clarity” OR “visual clarity”	"Endoscope” OR “laparoscope” AND “lens” AND “clean”
EMBASE	As above	As above
Cochrane registry	As above	As above

### Study selection

Inclusion and exclusion criteria were developed to assist with the identification of eligible studies for review (Table 3). The inclusion criteria comprised: research of primary quantitative design, publication in the past ten years (January 2009–December 2019), exposed to journal peer-review, text published in English language and reporting of intervention, context and outcome data relevant to the research question. No restrictions were placed upon study setting as generalisability was not fundamental to the review’s aims and objectives. Articles were not restricted by type of quantitative design, as the review sought to summarise all relevant evidence for the academic and clinical communities. The exclusion criteria included: research of secondary or primary qualitative design, publication prior to January 2009, lack of journal peer-review, text unavailable in English language and reporting of intervention and outcome data deemed irrelevant to answering the research question.

**Table 3:** Inclusion and exclusion criteria

Characteristic	Inclusion criteria	Exclusion criteria
Research design	Primary quantitative	Secondary reviews
		Primary qualitative
Publication date	January 2009–December 2019	Before January 2009
Language	English	Non-English
Journal peer-review	Yes	No
Setting	No restriction	-
Intervention	Methods/techniques/devices designed to clean endoscopic lenses	Interventions that did not involve cleaning of endoscopic lenses
Context	Minimally invasive abdominopelvic surgery	Open surgery
Outcomes	Optical clarity	Outcomes unrelated to optical clarity

### Data extraction and analysis

To avoid or attenuate the risk of data extraction errors that have previously compromised the credibility of several published systematic reviews, data from eligible articles was extracted by the utilisation of systematic pro formas developed and provided by the Cochrane Collaboration in their Handbook for conducting systematic reviews and meta-analyses [6, 7]. The analysis of data concerning the efficacy of endoscopic lens cleaning methods was considered for merging and meta-analysis, although inter-study heterogeneity was marked, and thus, meta-analysis was not possible, and data was analysed using narrative synthesis.

### Quality assessment

Considering the various research designs of informing studies, the critical appraisal skills programme (CASP) frameworks were adopted and used to inform judgements about internal validity and overall methodological quality [8]. The CASP frameworks were completed for each eligible study and determinations of overall quality were based upon the number and subjective impact of any systematic biases detected. Overall quality was rated as low, moderate or high risk of bias, which was based on the presence of 1–2 biases of low impact, 3–4 biases of moderate impact and > 4 biases with high impact, respectively. The critical appraisal process was conducted by the principal author and reviewed independently by their supervisor.

IRB approval was not required for the production of this paper.

## Results

### Search results and eligibility assessments

**Fig. 1:**
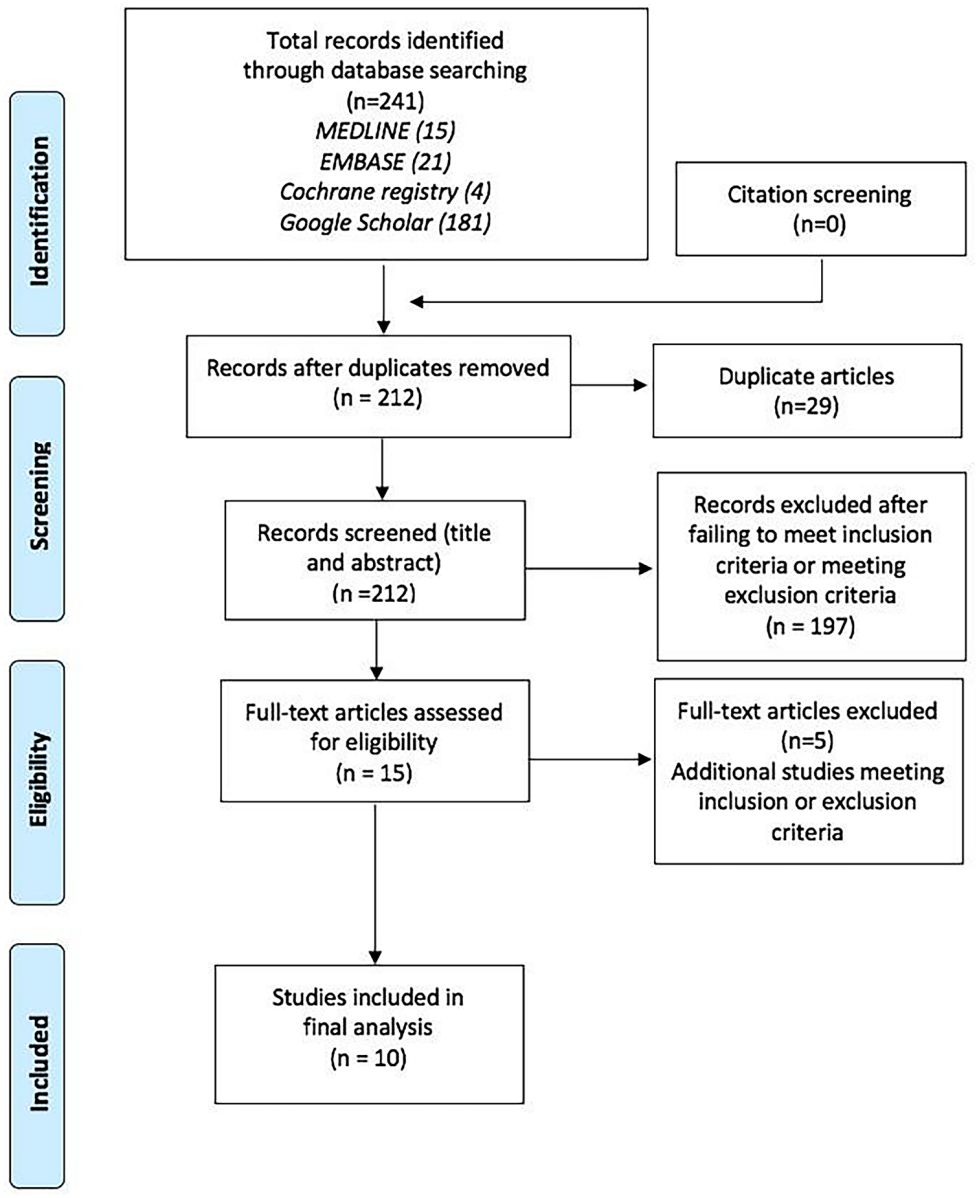
PRISMA diagram with a summary of the search results, filtering processes and eligibility determinations

A summary of the search results, filtering processes and eligibility determinations is provided in the PRISMA diagram in Fig. 1. Following the searching of MEDLINE, EMBASE, the Cochrane registry and Google Scholar, a total of 202 studies were retrieved, which included four duplicates that were subsequently removed from any further filtering and eligibility considerations. The titles and abstracts of the remaining 198 articles were screened for potential eligibility through application of the inclusion and exclusion criteria, which led to the exclusion of 183 studies. The residual 15 studies were further reviewed in their full texts for eligibility, which led to the further exclusion of five articles that failed to meet the inclusion criteria. Therefore, 10 studies were considered eligible for inclusion in this review.

### Study characteristics

#### Design

Eligible studies were found to have adopted various research designs to evaluate the effect of endoscopic lens cleaning/defogging methods and devices, which included four randomised controlled trials involving human participants [9–12], one simulated randomised controlled study [13], one prospective observational study [14] and four experimental studies/laboratory simulations [15–18]. Based on the guidance within the Grading of Recommendations, Assessment, Development and Evaluations (GRADE) criteria, the strength of research evidence should be considered in view of robust appraisal findings and specific biases or methodological issues such processes elicit [19]. The GRADE approach is important as traditional evidence pyramids, that tended to rank the quality of evidence in accordance with design, has become redundant as randomised controlled trials for example may derive evidence of low to high quality depending upon the extent and rigour of methods employed [20]. Similarly, observational studies that are inferred to derive low to modest levels of evidence by evidence pyramids can actually derive high quality evidence and thus, GRADE is appropriate for considerations of evidence strength and quality in this review [19]. Thus, despite the varied design of research studies included in this review, the critical appraisal judgements of each study were critical to informing the overall strength of evidence derived.

### Setting and participants/experimental models

Studies were conducted across various settings, including the western nations of the Netherlands [11], the United States [10, 13–15], Australia [17] and France [9], and non-western countries, including Korea [12] and Japan [16, 18]. Among studies involving human subjects, randomised trials [9–12] recruited participants using random sampling, while the prospective study [14] recruited subjects using consecutive sampling. Random sampling is considered the most effective sampling technique as it is almost always able to achieve balancing of known and unknown confounding variables of subjects between intervention and control groups, and thus, minimises the risk of confounding bias [21]. The consecutive sampling technique used by Drysch, Schmitt [14] was appropriate for the local operative context as exposure to the endoscopic lens systems was dictated by device changeover at a set time point; however, this approach is more susceptible to selection and confounding bias as the authors failed to measure baseline characteristics to assess for balancing of variables [21]. Patients included in the former studies were defined as follows: adults aged >  = 18 years and scheduled for gynaecological laparoscopic surgery [9, 12], adults aged >  = 18 years and scheduled for laparoscopic Nissen or Toupet fundoplication [10], patients undergoing laparoscopic partial nephrectomy, radical nephrectomy, nephrouretectomy or pyeloplasty [14] and patients undergoing laparoscopic donor nephrectomy [11].

Among experimental/simulated studies, various models were employed to assess the efficacy of endoscopic lens cleaning/defogging, which included; a 18.5 L watertight survival container and a porcine model [15], a surgical laparoscopic training mannikin [16], a plastic eight litre container partially filled with water to create peritoneal-like conditions [17], an insulated glass container [13] and a dark box containing artificial flowers [18]. Notably, only two of these studies [15, 16] utilised a representative model of the intra-abdominopelvic cavity and thus the efficacy reported among the other studies may hold little applicability to actual surgical contexts. The sample sizes for each study have been summarised in Table 4.

**Table 4:** Study matrix

Study and setting	Design	Subjects/model (N: sample size/trial repetitions)	Intervention/exposure	Control/comparator	Main limitations	Overall quality (risk of bias)	Applicability to surgical context
Bendifallah, Salakos [9] France	Prospective randomised controlled trial	Adults aged > = 18 years and scheduled for gynaecological laparoscopic surgery N = 104	FloShield air system involving carbon dioxide insufflation	Water with povidone iodine solution	Performance bias	Moderate	Moderate
					Detection bias		
Calhoun and Redan [15] United States	Experimental study	An 18.5 L watertight survival container N = 25	A prototype device with the ability to insufflate carbon dioxide	None employed	Performance bias	Low	Low
					Detection bias		
					Low sample size		
					Experimental design		
Cassera, Goers [10] United States	Prospective randomised controlled trial	Adults aged > = 18 years and scheduled for laparoscopic Nissen or Toupet fundoplication N = 40	EndoClear device involving cleaning fabric for lens wiping intra-abdominally	Standard endoscopic device or conventional gauze wiping of contaminated lens	Performance bias	Low	Moderate
					Detection bias		
					Low sample size		
Drysch, Schmitt [14] United States	Prospective observational study	Patients undergoing laparoscopic partial nephrectomy, radical nephrectomy, nephrouretectomy or pyeloplasty N = 40	Fluid Warming System involving heating of laparoscope-introduced fluids	Clearify Visualisation System involving heating of laparoscope	Performance bias	Low	Moderate
					Detection bias		
					Lack of randomisation		
					Low sample size		
Kobayashi, Kakuda [16] Japan	Experimental study	A surgical laparoscopic training manikin N = 280	Endowiper device with a tightly wrapped cotton gauze	Small and large gauze wiping and wiping with a sterilised swab	Performance bias	Low	Low
					Detection bias		
					Experimental design		
Manning, Papa [17] Australia	Experimental study	A plastic eight litre container partially filled with water to create peritoneal-like conditions N = 30	Surfactant solutions of FRED and Ultra-Stop and chlorhexidine and betadine solutions	Laparoscope warming	Performance bias	Low	Low
					Detection bias		
					Low sample size		
					Experimental design		
Merkx, Muselaers [11] Netherlands	Prospective randomised controlled trial	Patients undergoing laparoscopic donor nephrectomy N = 50	Heating of sterile water	ResoClear surfactant solution	Performance bias	Moderate	Moderate
					Detection bias		
					Low sample size but sufficient power		
Palvia, Gonzalez [13] United States	Simulated prospective randomised controlled trial	An insulated glass container N = Unclear	Surfactant solution of FRED, chlorhexidine, warmed saline and glove warming	Absence of any defogging techniques	Performance bias	Low	Low
					Detection bias		
					Unclear sample size and power		
					Experimental design		
Song and Lee [12] Korea	Prospective randomised controlled trial	Adults aged > = 18 years and scheduled for gynaecological laparoscopic surgery N = 96	Multiple interventions including heated saline, Ultra-Stop surfactant solution and chlorhexidine	A lack of surfactant solution or gauze wiping or for contaminated lenses – manual wiping of lens by a scrub nurse	Performance bias	Moderate	Moderate
					Detection bias		
Tatsuki, Yokobori [18] Japan	Experimental study	A dark box containing artificial flowers N = Unclear	Composite novel device of air and water insufflation	A simple gauze wiping manoeuvre	Performance bias	Low	Low
					Detection bias		
					Unclear sample size and power		
					Experimental design		

### Interventions/exposures and comparators

Among human studies, the endoscopic lens cleaning methods/devices employed comprised; the FloShield Air System involving carbon dioxide insufflation [9], the EndoClear device involving cleaning fabric for lens wiping [10], the Fluid Warming System involving heating of laparoscope-introduced fluids [14], heating of sterile water [11] and multiple interventions including heated saline, Ultra-Stop surfactant solution and chlorhexidine [12]. The comparators/controls for these studies comprised; water with povidone iodine solution [9], standard endoscopic device or conventional gauze wiping of contaminated lens [10], the clearify visualisation system involving retraction of the scope followed by insertion into the device for automatic mechanical cleaning [14], ResoClear surfactant solution [11] and a lack of surfactant solution or gauze wiping for contaminated lenses–manual wiping of lens by a scrub nurse [12]. While there was no apparent contamination of the interventions and controls across all human studies, there may have been significant variance in the efficacy of the interventions/controls employed as a result of the laparoscopes being used by different surgical operators, which can be termed observer or detection bias [22]. In addition, the visible nature of the interventions and comparators employed prevented the ability to blind observers and thus, all the former studies have a risk of performance bias [23].

Among experimental studies, the endoscopic lens cleaning methods/devices employed comprised; a prototype device with the ability to insufflate carbon dioxide [15], the Endowiper device with a tightly wrapped cotton gauze [16], surfactant solutions of FRED and Ultra-Stop and chlorhexidine and betadine solutions [17], surfactant solution of FRED, chlorhexidine, warmed saline and glove warming [13] and a composite novel device of air and water insufflation [18]. A comparator was not employed by Calhoun and Redan [15] but were in other studies as follows: small and large gauze wiping and wiping with a sterilised swab [16], laparoscope warming [17], absence of any defogging techniques [13] and a gauze wiping manoeuvre [18]. Notably, due to the lack of blinding and experiments conducted by multiple operators, these studies also observe the same risk of detection and performance biases as the ones mentioned in the human studies. [23].

### Outcome measures

Studies included in this review employed various means to measure the cleaning efficacy of the interventions/comparators, which included; number of laparoscope removals, cleaning duration and optical clarity determined using Likert-type scales that were rated subjectively by each operator [9, 10, 13], subjective presence or absence or degree of fogging and obstructed view without the use of a Likert-type scale [12, 15–18] and the simple frequency of fogging events [11, 14]. Various other outcomes were measured and are discussed in the outcomes section of this review. Notably, outcomes that were assessed using Likert-type scales are likely to have generated more reliable findings given that these help to account for significant variances in subjectivity and thus, may have minimised the risk of measurement bias [24]. There was no risk of attrition across all studies in this review.

### Quality summary

Based on the detection and impact of various systematic biases, studies in this review could only be rated as being low to moderate in methodological quality. However, studies involving human participants can be generalised to other populations undergoing laparoscopic abdominopelvic surgery, although the external validity is compromised for all experimental studies. In this regard, the excess heterogeneity across studies in regard to the type of laparoscopic procedure being performed is likely to influence the risk and degree of laparoscopic lens decontamination, as well as the efficacy of cleaning methods and the risk of recurrent decontamination. Thus, judgements about external validity may be best considered on a study-by-study basis. A summary of each study including its limitations is provided in Table 4.

## Discussion

In summary, this systematic review aimed to evaluate and summarise the current evidence base pertaining to the effectiveness of methods and devices designed to clean endoscopic lenses used for laparoscopic abdominopelvic surgery. The evidence was synthesised under two main themes of (1) Standard or readily available cleaning devices/methods and (2) Novel cleaning methods/devices. among conventional and readily available methods, the most effective approaches involved heated sterile water, heating of laparoscope lenses, and surfactant solutions, including FRED and Ultra-Stop. Novel devices and methods were more effective than controls, which included lens wiping systems and air and carbon dioxide flow systems. While the former surgical techniques were consistently associated with superior lens cleaning ability and/or defogging capability and subsequent optical clarity of images within the surgical field, no methods conferred any meaningful effects upon other clinically important outcomes, such as operative time, costs, complication rates and length of stay. This suggests that decision making concerning the selection of lens cleaning method/device should suit the preferences of the instrument operator or the responsible surgeon.

The gradual or sudden loss of the surgical field during laparoscopy is a common and well-known issue generating frustration among surgeons and interrupting cognitive and tactile performance and operative flow [25, 26]. Secondly, insufficient optical clarity of the visual field can be markedly hazardous to both the safety and outcomes of patients undergoing minimally invasive surgery given that tactile feedback and the extent of the surgical field is already restricted when compared to open surgery and thus, the management of complications, such as haemorrhage, may be delayed as a result of poor or impaired detection [27, 28]. Although the fogging of endoscope lenses is generally inferred to result from contamination of the lens due to matter within the locally operative anatomy, including debris, blood and surgical smoke, fogging in its truest sense usually results from condensation of liquid droplets due to the presence of surrounding heat and moisture [25, 29]. As fogging due to condensation is the most frequently encountered problem affecting visual clarity in endoscopic surgery, methods and techniques used to counter this problem are likely to be most useful in elective surgical settings [30]. Furthermore, when it came to looking at the impact the surgical procedure has on lens contamination, it was reported that surgeons spend about 3% of their time during laparoscopic Nissen fundoplication’s on cleaning the endoscope lens. [10] Schoofs and Gossot [31] found that soiling of the endoscope lens during thoracoscopic procedures was considered troublesome by 68% of the surgeons. Alternately, a study conducted by Abbitt et al. [32] was able to prove no statistically significant differences in the mean number of times the laparoscope was withdrawn between general surgery cases and gynaecological surgery cases, as well as no statistically significant difference in the mean length of time the laparoscope was withdrawn between general surgery cases and gynaecological surgery cases. However, cases that required a longer surgical time (exceeding 30 min) had a significantly higher number of times the laparoscope needed to be withdrawn for cleaning as compared to shorter cases (< 30 min).

In this review, the majority of studies demonstrated that gas insufflation and surfactant solutions were highly effective in clearing condensation on lenses, while cleaning of lenses contaminated by blood or debris also benefitted from physical wiping of the lens surface. Scientific theory states that condensation of endoscopic lenses is influenced by differences in humidity and temperature of the laparoscope and the intra-abdominal/pelvic environment, suggesting that a degree of fogging may not always be preventable or resolvable but this was disproven among studies reported in this review [33]. Indeed, the preheating of laparoscopic instruments and lenses or lens solutions to temperatures above the dew point temperature of the intra-abdominopelvic environment prevented condensation, which was apparent in the outcomes reported by Drysch, Schmitt [14], Merkx, Muselaers [11], Song and Lee [12] and Manning, Papa [17]. The positive effect of laparoscope heating has been previously supported by the anecdotes of Brown, Inocencio [34] who found that the use of a water bath set at 50 °C to maintain warmth of the inserted instrument and lens resulted in significant reductions in peri-operative fogging events during a 5 year observation period. However, it is apparent that this preheating technique can attenuate over time where the cooling of the laparoscope and any lens solutions may fall to a temperature below the dew point of the intra-abdominopelvic environment where condensation and fogging then ensues, although this is most likely to arise when the lens is removed from the surgical cavity for clearing of debris or blood as it exposed to the colder operating room temperatures [35]. This may imply that surfactant solutions could be more effective at preventing lens fogging, although this was only supported by Manning, Papa [17] and was disputed by Song and Lee [12], Merkx, Muselaers [11], Palvia, Gonzalez [13] and Drysch, Schmitt [14].

The anti-fogging effects of surfactant solutions, such as FRED and Ultra-Stop, can be attributed to the adsorption of the solutions onto lens surfaces, which in turn, modify the free energies of interacting molecules and lower the surface tension that effectively permits the scattering of water and other liquid droplets [36]. However, not all evaluations of surfactant solutions resulted in a desirable level of defogging and visual field clarity, although this is likely to reflect the relatively poor long-term stability of surfactant compounds, which can impair the degree of surface tension imparted upon lenses and therefore, the adherence of liquid droplets and the degree of fogging [37]. In addition, it has been reported that different surfactant solutions observe varying properties when exposed to specific temperature ranges, which can effectively impair the solubilisation of the compounds [36] and this may have accounted for variances between FRED and Ultra-Stop as reported by Manning, Papa [17], Palvia, Gonzalez [13] and Song and Lee [12]. Authors among the wider literature support the efficacy of surfactant solutions in preventing lens fogging but have failed to conduct quantitative analyses [38, 39].

The anti-fogging efficacy of surfactants may, like preheating techniques, dissipate over time as a result of laparoscope withdrawal and manual lens cleaning of debris and/or blood, which can remove and/or impair binding of surfactant compound [40]. Notably, the insufflation of air and carbon dioxide offer a solution to this problem by negating the need for endoscope withdrawal and indeed, the positive effect upon lens defogging, mediated by the pressure exerted upon lenses from insufflated gases, was reported by Calhoun and Redan [15] and Bendifallah, Salakos [9] in this review. Few other studies have evaluated the effect of gas insufflation techniques. In one study, Schurr, Bablich [41] found that carbon dioxide insufflation to clear endoscopic lenses from fogging and contamination was highly effective and efficient, although the outcomes were not measured against an active comparator. In another study, Farley, Greenlee [42] conducted a double-blind randomised trial to investigate the effect of standard versus warmed and humified carbon dioxide insufflation upon lens fogging in patients undergoing laparoscopic cholecystectomy. The authors found that there were no significant differences in fogging events between methods. However, the trocars required to insufflate gases to clear endoscopic lenses are generally of 12 mm in diameter and thus, may not be preferable among or convenient to surgeons performing minimally invasive procedures [40].

Despite the various methods evaluated in this review, there were no significant differences in complication rates between the intervention and control groups. This could mean that the problem of endoscopic lens fouling/fogging and its cleaning is one that may only have minor implications for patients, the operation, the surgical operators and other surgical and anaesthetic outcomes. In contrast, it could also imply that the interventions of lens cleaning/defogging methods and devices are not as efficient as they could be, especially as most of them could not resolve all lens contamination events without interrupting the flow of the surgery. However, positive effects upon the former outcomes could have been missed due to bias and other methodological issues among the informing evidence included in this review. Indeed, much of the evidence focussed upon primary outcome measures related to laparoscopic lens fouling or fogging events and thus, failed to consider patient and operative outcomes. Wider research has shown that laparoscopic lens fogging and removal of laparoscopes during surgery can be associated with significant increases in operative time and blood loss, although the effect sizes reported were small and thus, it is not clear whether reductions in lens fogging or fouling would lead to meaningful improvements in operative time and blood loss in routine practice [27, 43].

The findings of this review are subject to a number of other limitations, which are discussed to demonstrate objectivity and to provide a context for reflection. Firstly, an evidence-based strategy was developed to permit extensive literature searching in order to ensure the identification of all relevant studies to address the research question, although pertinent articles may have been excluded due to the restriction criteria employed and this could have affected the outcomes reported. On reflection, this is a risk that can never be completely managed and eliminated given that articles are continually added to the evidence base and considering that database indexing procedures are never 100% accurate. In this regard, we discovered that citation screening was a highly effective option in confirming the risk of missing pertinent studies and indeed, no additional studies were identified through this process, suggesting that all relevant articles were captured and included in this review. Secondly, the heterogeneity evident across included studies prevented the ability to conduct meta-analysis, which is considered the most rigorous and objective means to collectively analysing outcome measures of an intervention or exposure. However, the alternative of narrative synthesis was sufficient in describing and reporting the most important outcomes and this was perceived to have successfully addressed the research question.

## Conclusion

The ability to achieve and sustain a high level of optical clarity during minimally invasive laparoscopic surgery is essential to optimal and efficient surgical performance and potentially, patient safety. The findings of this systematic review demonstrated that a range of endoscopic lens cleaning methods and devices can be used to achieve sufficient optical clarity of the laparoscopic surgical field through either preventing lenses from fogging and/or facilitating the inter-operative cleaning of fouled lenses. Our study found no difference in outcomes between the intervention and the control groups. In order to truly enhance the lens cleaning process in a way that could potentially improve outcomes, more research and development efforts should focus on designing universal, portable, low energy, low cost, and high efficiency technology capable of removing all lens contaminants without interrupting the flow of the surgery. Ideally one that is autonomous, automatic, and compatible with established surgical instruments.

### Recommendations for future research

Overall, there was a scarcity of primary evidence having explored the efficacy of cleaning methods for laparoscopic lenses decontaminated during abdominopelvic surgery and thus, it is important that future research conducts more related studies but importantly, seeks to account for the limitations of the evidence evaluated herein. There was particular heterogeneity in regard to the type of laparoscopic procedure being performed and laparoscopic equipment and thus, it is important for future research to utilise similar measures to help validate the findings of the primary evidence base. This could provide more certainty in the effects reported and the external validity of evidence herein. Moreover, it would be useful for future research to ensure homogeneity of laparoscopic operators, in order to reduce any bias associated with inter-operator performance. Finally, novel surgical technologies that have the potential to transform the future of surgery are actively being developed and translated to clinical settings, especially in the fields of laparoscopy, robotic surgery, and surgical imaging. Having an optimal vision is fundamental for such technologies to perform well. Repeated loss of visualisation during keyhole surgery is a cumbersome inefficiency that needs to be addressed going forward, as it has multiple negative implications on surgical performance, patient safety, time, and cost. With millions of laparoscopic surgeries performed every year, the global cumulative effect of laparoscopic lens contamination cannot be neglected anymore.
